# Supplementary material for: Restoration of Osteogenesis by CRISPR/Cas9 Genome Editing of the Mutated COL1A1 Gene in Osteogenesis Imperfecta
Source: J Clin Med. 2021 Jul 16;10(14):3141. doi: 10.3390/jcm10143141 (PMC8307903; doi:10.3390/jcm10143141)
Supplement: Supplementary file 1 [file jcm-10-03141-s001.zip › jcm-1271195-supplementary.pdf]

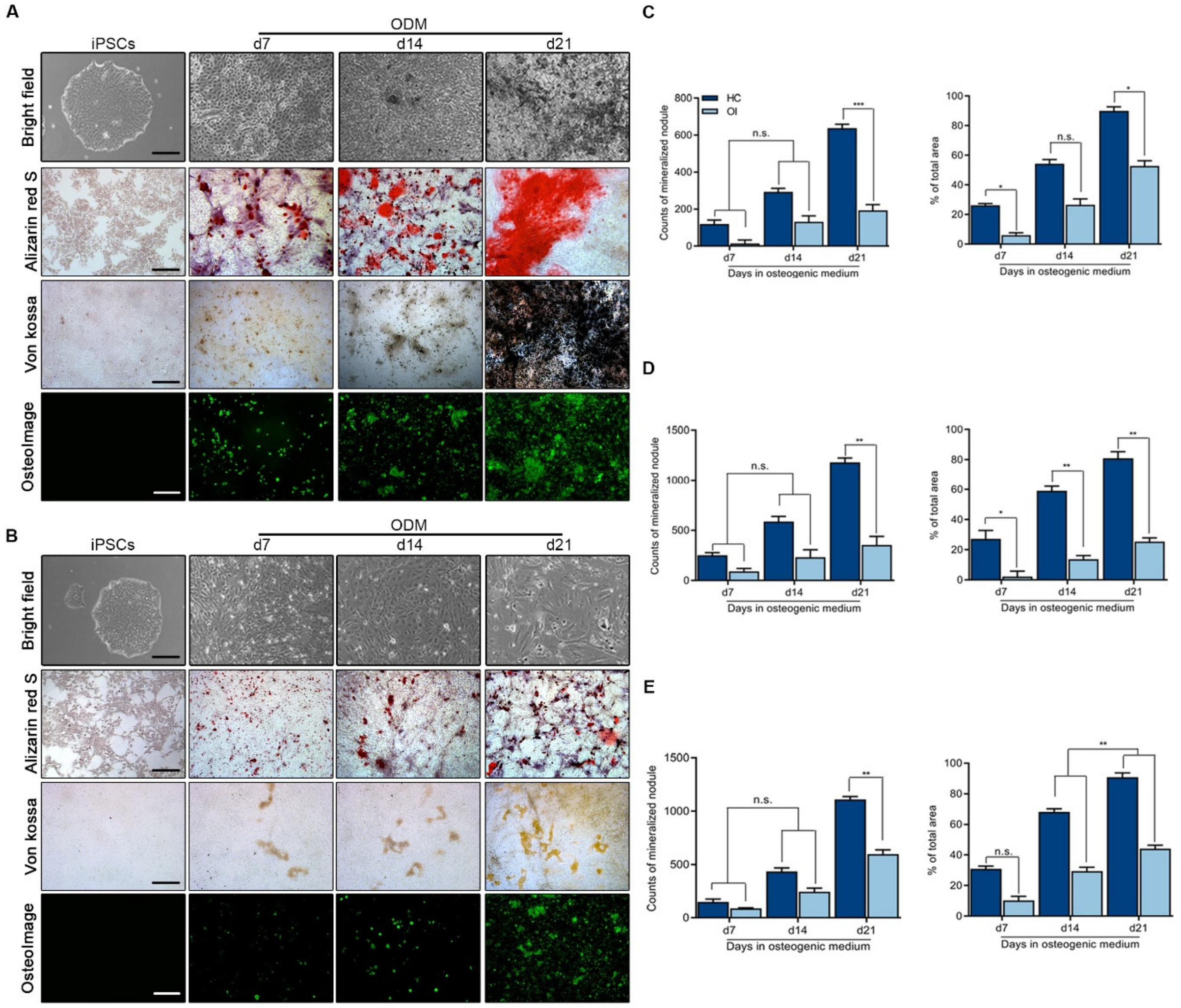

Figure S1. Decreased mineralized nodule formation of OI-iPSCs

(**A**) HC-iPSCs cultured in osteogenic differentiation media for 7, 14, and 21 days with Alizarin Red S and von Kossa to visualize calcium mineral deposition. The amount of hydroxyapatite was measured using the OsteoImage mineralization assay, in which a fluorescent green dye binds to the hydroxyapatite portion of the mineralized matrix. Scale bar, 100 µm. (**B**) OI-iPSCs cultured in osteogenic differentiation media for 7, 14, and 21 days with Alizarin Red S and von Kossa to visualize calcium mineral deposition. The amount of hydroxyapatite was measured using the OsteoImage mineralization assay, in which a fluorescent green dye binds to the hydroxyapatite portion of the mineralized matrix. Scale bar, 100 µm. Quantitative measurements of (**C**) Alizarin Red S and (**D**) Von Kossa using ImageJ software. (**E**) The amount of hydroxyapatite was measured using the OsteoImage mineralization assay, and quantified using the ImageJ software. Data are means ± SEM. *** $p<0.001$ vs.OI, ** $p<0.005$ vs.OI, * $p<0.01$ vs.OI, indicated statistical significance (Unpaired t-test).

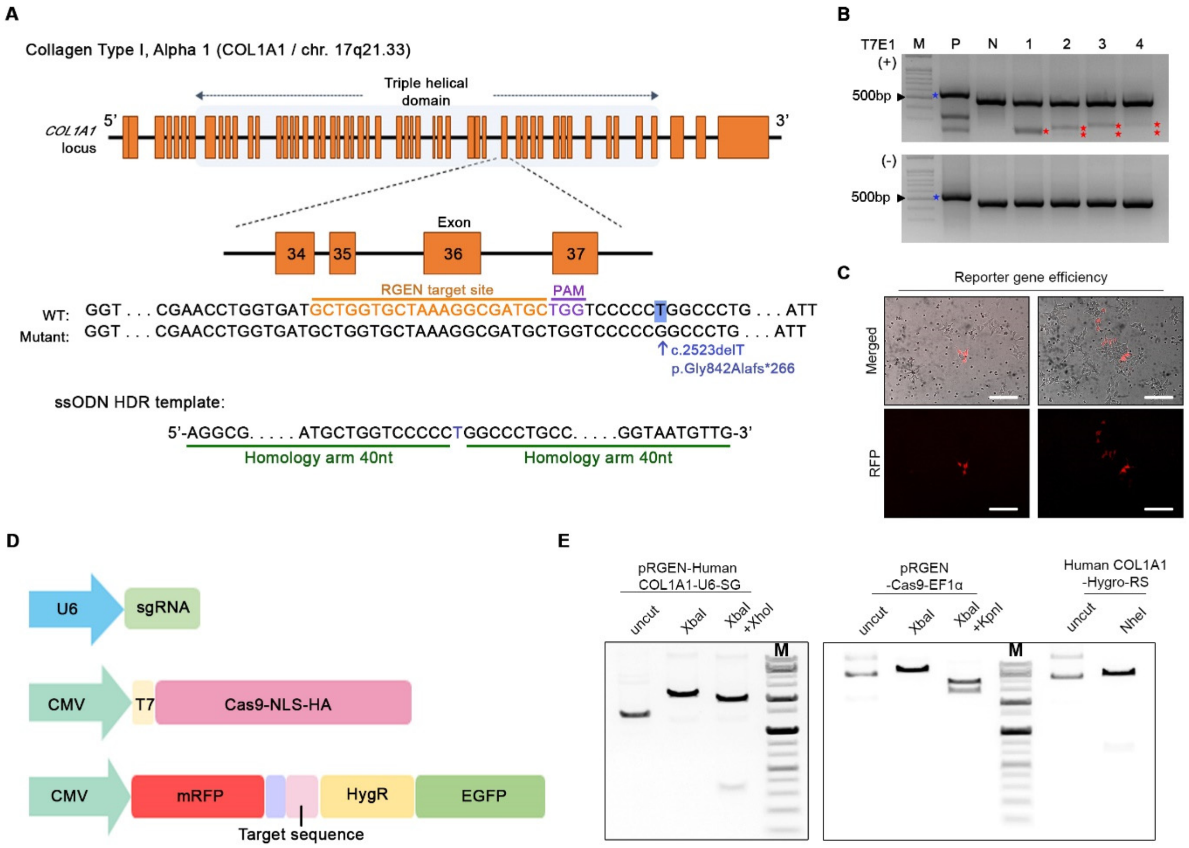

Figure S2. Selection of the most efficient RGENs and reporter gene through screening methods

(**A**) Schematic illustration of RGEN targeting sequences on the 36 exon of COL1A1 gene. COL1A1 was found at q21–q25 bands of chromosome 17, where c.2523delT mutations in exon 36. ssODN, was designed as a 48bp of homology arms flanking each side of the mutation site of COL1A1 gene. The 20-bp target sequence of RGEN target site is shown was highlighted in yellow. The PAM sequence is shown was highlighted in pink. (**B**) Mismatch Cleavage Assay was performed using T7 Endonuclease I. T7E1 analysis results showing cut products on the left. The graph on the right is the expected cut pattern. P; positive control, N; negative control, Lane 1 RGEN1, lane 2 RGEN2, lane 3 RGEN3 and lane 4 RGEN4. (**C**) Evaluation of transfection efficiency of RFP reporter gene in HEK 293T cells. Scale bar, 100 μm. (**D**) Overview of the CMV-Cas9 and RGEN constructs used in this study. (**E**) Representative gel electrophoresis pictures show restriction digestion patterns after cloning using restriction enzymes.

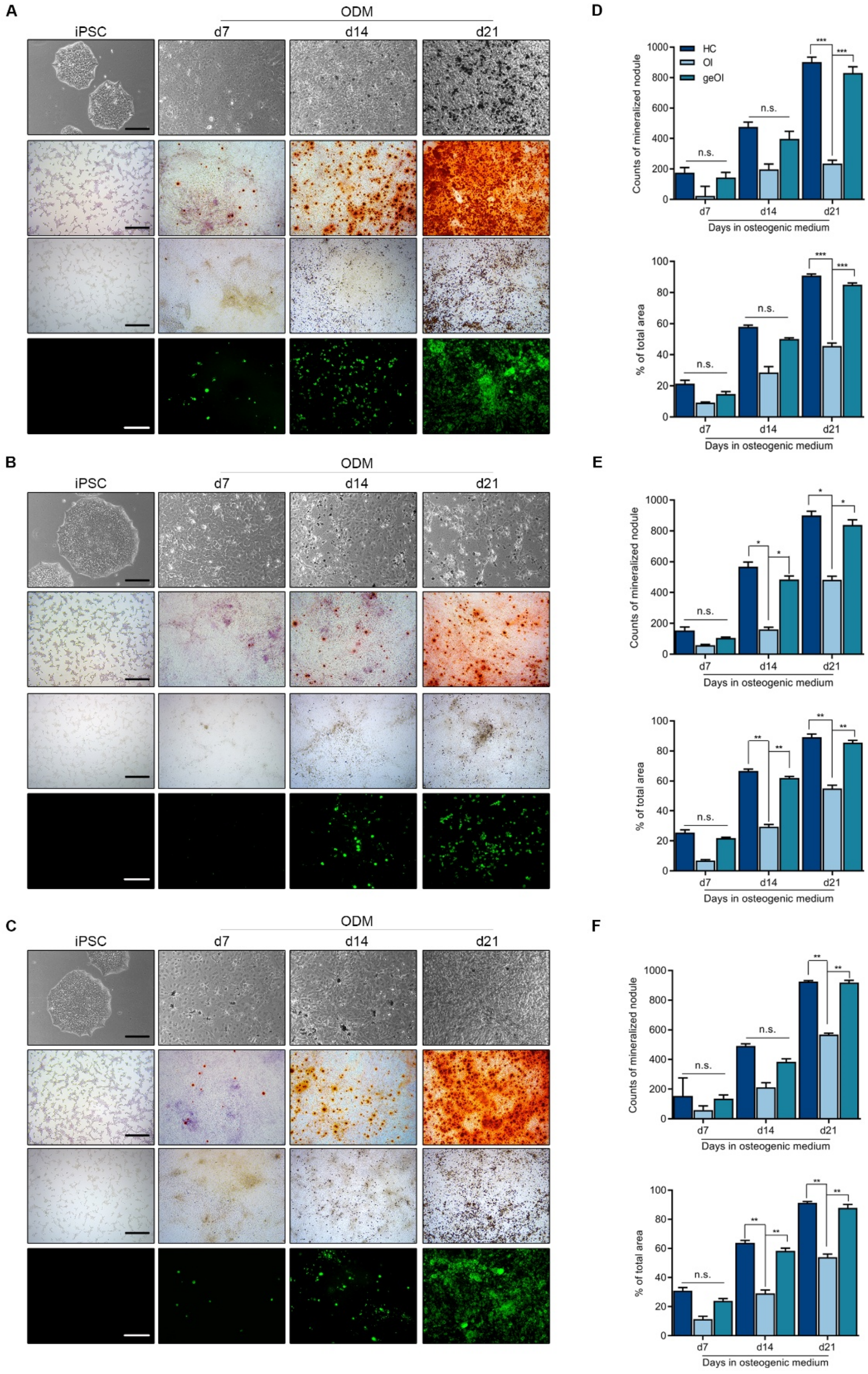

Figure S3. Increased mineralized nodule formation of editOI-iPSCs

(**A**) HC-iPSCs were cultured in osteogenic differentiation media for 7, 14, or 21 days with Alizarin Red S and Von Kossa to confirm calcium mineral deposition. The amount of hydroxyapatite was measured using the

OsteoImage mineralization assay, in which a fluorescent green dye binds to the hydroxyapatite portion of the mineralized matrix. Scale bar, 100 μm. (**B**) OI-iPSCs were cultured in osteogenic differentiation media for 7, 14, or 21 days with Alizarin Red S and Von Kossa to confirm calcium mineral deposition. The amount of hydroxyapatite was measured using the OsteoImage mineralization assay, in which a fluorescent green dye binds to the hydroxyapatite portion of the mineralized matrix. Scale bar, 100 μm. (**C**) editOI-iPSCs were cultured in osteogenic differentiation media for 7, 14, or 21 days with Alizarin Red S and Von Kossa to confirm calcium mineral deposition. The amount of hydroxyapatite was measured using the OsteoImage mineralization assay, in which a fluorescent green dye binds to the hydroxyapatite portion of the mineralized matrix. Scale bar, 100 μm. Quantitative measurements of (**D**) Alizarin Red S and (**E**) Von Kossa using ImageJ software. (**F**) The amount of hydroxyapatite was measured using the OsteoImage mineralization assay, and quantified using the ImageJ software. Data are means ± SEM. *** $p<0.001$ vs.OI, ** $p<0.005$ vs.OI, * $p<0.01$ vs.OI, indicated statistical significance (Unpaired t-test).
